# Controlling the Properties of Poly(3-hydroxybutyrate) through Lignin-Containing Organogels

**DOI:** 10.1021/acs.biomac.6c00394

**Published:** 2026-05-14

**Authors:** Alexandros E. Alexakis, Unnimaya Thalakkale Veettil, Minna Hakkarainen, Mika H. Sipponen

**Affiliations:** † Department of Chemistry, 7675 Stockholm University, Stockholm 10691, Sweden; ‡ Department of Fibre and Polymer Technology, KTH Royal Institute of Technology, Teknikringen 58, Stockholm 100 44, Sweden

## Abstract

Poly­(3-hydroxybutyrate) (PHB) is a biodegradable polyester with a slow hydrolytic degradation rate linked to its high crystallinity. Here, PHB-lignin organogels were prepared using the bio-based solvent Cyrene to investigate how processing and lignin type, softwood (SKL) or hardwood (HKL) kraft lignin, affect structure and water stability. Freeze-dried organogels (up to 50 wt % lignin) were analyzed by FTIR spectroscopy, SEM, DSC, and XRD. Organogel formation reduced PHB crystallinity by about 10%, while lignin incorporation further decreased it by up to 84% (SKL) and 73% (HKL), lowering melting enthalpy and promoting cold crystallization. Although HKL caused slightly earlier crystallization suppression during cooling and smaller crystallites at low loadings, both lignin types produced similar crystallinity reductions and degradation behavior. Water exposure increased the mass loss compared with neat PHB, consistent with decreased crystallinity. Solvent extraction enabled partial lignin recovery and PHB recrystallization. Overall, a processing-based approach to tuning PHB crystallinity and degradation in lignin-containing biocomposites is demonstrated.

## Graphical abstract

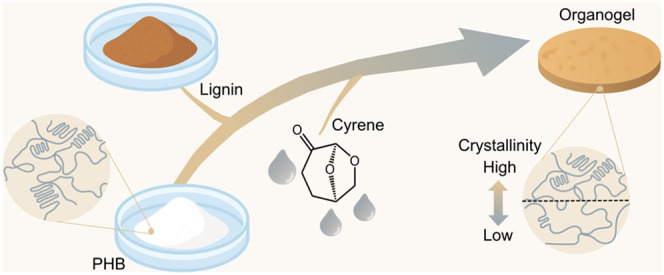

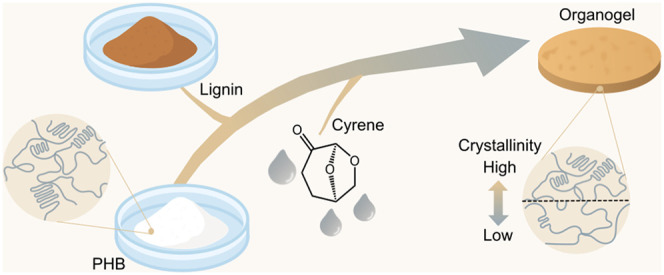

## Introduction

Poly­(3-hydroxybutyrate) (PHB) is the most studied member of the polyhydroxyalkanoate (PHA) family, valued for its excellent barrier properties, biodegradability, biocompatibility, and mechanical strength.^1,2^ These features are relevant for a wide range of applications in packaging, biomedical, and agriculture.^3–6^ However, despite this broad application potential, global production of PHB remains relatively small, on the order of ∼100,000 tonnes per year (which is expected to increase to almost 1 M tonnes per year by 2029),^7^ compared with conventional plastics produced altogether at about four hundred millions of tonnes annually. This underscores both scale limitations and industrial challenges for broader adoption.^8^ Moreover, despite being biodegradable, PHB often shows slow hydrolytic degradation rates, especially under many environmental conditions.^9–11^ The rate of degradation is, to a large extent, controlled and limited by the high crystallinity (typically ∼40–90%)^2,12,13^ since hydrolysis and microbial attack tend to start in amorphous regions, while crystalline lamellae are highly resistant.^10,14^

A common way to modify crystallinity is through polymer blending, which affects chain mobility, nucleation, and crystalline content.^12^ Blends with polymers such as poly­(lactic acid) (PLA),^15^ poly­(ε-caprolactone) (PCL),^16,17^ cellulose derivatives,^18,19^ and starch^20,21^ have been shown to significantly decrease the crystallinity of PHB. More recently, attention has shifted toward blending with biological macromolecules such as lignin, which itself lacks crystallinity and can disrupt the spherulitic growth of PHB by forming hydrogen bonds or acting as a steric barrier to chain packing.^22–25^ However, the intensity of these effects depends primarily on the chemical structure, molecular weight distribution, and compatibility of the specific lignin type with the PHB matrix.

The structure of lignin is naturally heterogeneous, primarily composed of *p*-coumaryl alcohol, coniferyl alcohol, and sinapyl alcohol, with varying ratios found in softwood and hardwood lignins and further structural modifications taking place during different pulping processes.^26,27^ Specifically, softwood kraft lignin (SKL), which is rich in guaiacyl units, generally has a more condensed, cross-linked structure, while hardwood kraft lignin (HKL) contains more syringyl units with fewer condensed linkages, influencing solubility and polymer compatibility.^28,29^ These chemical differences significantly influence lignin’s capacity to interact with PHB by impacting crystallization kinetics, hence affecting material properties. Blends of PHB or other PHAs with lignin derivatives have been prepared by using melt extrusion, solvent casting, or reactive processing. For instance, thermophysical studies of PHB/lignin blends have shown that lignin accelerates crystallization and reduces overall crystallinity compared to pure PHB.^23,25^ Enhancements in thermal stability, antioxidant activity, and UV protection have also been noted alongside reductions in crystallinity, depending on lignin type and dispersion quality.^24,30^ Beyond physical blending, lignin has been used as a raw material for PHB biosynthesis, where aromatic monomers from lignin depolymerization are metabolized by engineered microorganisms to produce PHB, offering a promising integrated valorization pathway within lignocellulosic biorefineries.^31–33^ Nonetheless, melt-blended PHB/lignin systems often face limitations in lignin content (usually under 10–15 wt %) and aggregation due to poor compatibility, which further restricts the ability to tailor crystallinity and subsequent material properties.^12^

Recently, PHB organogels have emerged as an alternate processing approach, where physically entangled PHB chains form stable three-dimensional networks in organic solvents.^34–36^ These organogels provide opportunities for creating highly porous structures via freeze-drying, attracting interest, for example, in art restoration.^36^ However, combining PHB and lignin into organogels by sustainable processing with environmentally friendly solvents remains an unexplored area. Dihydrolevoglucosenone (cyrene), a biomass-derived dipolar aprotic solvent,^37^ shows potential for dissolving many polymers and is compatible with lignin. However, it has not yet been utilized for forming PHB–lignin hybrid organogels. Although some reports indicate that high lignin incorporation is achievable,^25^ there remains a lack of understanding regarding how processing methods, lignin type, and lignin content influence crystallinity and degradation behavior.

We anticipated that the addition of lignin during processing could be used to control the crystallinity of PHB, which could be further exploited to tailor the degradation behavior and properties. Therefore, we prepared PHB–lignin organogels in the green solvent Cyrene and subjected them to freeze-drying to produce hybrid structures with different lignin loadings up to 50 wt %. The effects of lignin type (softwood vs hardwood kraft lignin) and lignin content on molecular interactions, crystallinity, thermal behavior, and morphology were systematically examined. Additionally, the impact of these structural changes on the hydrolytic degradation was evaluated in a simple aqueous medium. This provides a new route for valorizing lignin in functional biopolymer materials.

## Experimental Section

### Materials

Polyhydroxybutyrate (PHB, ENMAT Y3000 from TianAn, lot number: EDS2364, selected material properties: specific gravity (1.25), yield stress (31–36 MPa), tensile strength (38 MPa), Young’s modulus (1600–2100 MPa)), Cyrene (Sigma-Aldrich), softwood kraft lignin (SKL, BioPiva100, purchased from UPM),^38^ and hardwood kraft lignin (a kind gift from Prof. Monika Österberg from Aalto University, Finland) were used as received.

### Organogel Preparation

PHB (0.8 g) was mixed with Cyrene (8 mL) and varying amounts of SKL or HKL (ranging from 5 to 50 wt % relative to the PHB amount) while maintaining a constant ratio between PHB and Cyrene in all formulations. The mixture was heated to 120 °C under constant stirring until the PHB melted (about 15–20 min).^35,36^ Then, the viscous mixture was poured into glass Petri dishes while still hot and allowed to cool at room temperature. The organogels were freeze-dried for further characterization. In the rest of this study, we use the following nomenclature for the samples: PHBx-SKL/HKLy, where x and y correspond to the wt % content of each component. For example, PHB60-SKL40 indicates that 40 wt % of SKL was used in this organogel. Additionally, the original PHB powder is denoted as PHB original, and the processed PHB organogel with Cyrene is denoted as PHB100.

### Fourier Transform Infrared Spectroscopy

FTIR data were collected by using a Varian 610-IR FTIR spectrometer. All samples were lyophilized before analysis, and were analyzed using 16 scans between 400 and 4000 cm^–1^.

### Scanning Electron Microscopy

SEM imaging was conducted using a JSM-IT 800 (JEOL Ltd.) and a secondary electron detector. The freeze-dried samples were cast onto carbon tape and sputtered with Au, using a JFC-1200 fine coater for 30 s.

### Differential Scanning Calorimetry

DSC was used to determine the thermal properties of the bio-based organogels, using a Netzsch DSC 214 Polyma instrument. The program included a heating ramp from 20 to 220 °C, an isothermal step at 220 °C for 5 min, a cooling ramp from 220 to −50 °C, an isothermal step at −50 °C for 5 min, and finally a heating ramp to 250 °C. The measurements were performed under a nitrogen atmosphere with a heating and a cooling rate of 10 °C/min. The crystallinity degree (*X*_c_^DSC^) was calculated based on eq 1

1 $$X_{c}^{DSC}=\frac{\left(\Delta H_{m}-\Delta H_{cc}\right)}{\Delta H_{m}^{o}\times {\;w}_{PHB}}\times 100$$

where Δ*Η*_m_ is the enthalpy of melting, Δ*Η*_cc_ is the cold crystallization enthalpy, *w*_PHB_ is the weight fraction of PHB in each formulation, and Δ*H*_mo_ (146 J/g)^12^ is the enthalpy of 100% crystalline PHB. All values reported are the average values of triplicates.

### X-ray Diffraction

Powder X-ray diffraction (XRD) patterns of the samples were obtained using a D8 Discover Diffractometer in reflection mode using Cu Kα radiation (λ = 1.5418 Å), with 2θ ranging from 5° to 85° with an increment of 0.01 and a rotation speed of 15 rotations per min. The crystallinity degree (*X*_c_^XRD^) was calculated by dividing the area of the crystalline peaks by the total area (amorphous and crystalline). The average crystallite size (*D*) was found using the Debye–Scherrer^39^ equation (eq 2)­

2 $$D=\frac{0.9\times \lambda }{d\times \cos\theta }$$

where λ is the wavelength (nm), *D* is the crystallite size (nm), *d* is the full width at half-maximum intensity of the peak (radians), and θ is the Bragg diffraction angle.

The lattice spacing (*hkl*) and crystal lattice parameters (*a*, *b*, and *c*) were calculated based on *D* of eq 2 and the known orthorhombic crystal structure of PHB.^40,41^ This was performed on known *hkl* indexes, namely, 020 and 110; therefore, the calculation of *a* and *b* was only possible due to the amorphic enhancement imposed by the presence of lignin. The results are listed in Table S1.

### Water Stability

Water stability was performed at ambient temperature in deionized water. Four samples, namely, PHB original, PHB100, PHB60-SKL40, and PHB60-HKL40, were selected to investigate the effects of the bio-based solvent and lignin type on the hydrolysis rate of the bio-based organogels. Briefly, powders between 20 and 60 mg of the formulations above were transferred to sealed vials containing 20 mL of deionized water. The water stability experiment was performed over 2 months in triplicate. The mass was recorded, and the residual mass after the water stability test was calculated by eq 3.

3 $$Residual\;mass\left(\%\right)=\frac{m_{d}}{m_{o}}\times 100$$

where *m*_d_ is the dry mass after the water stability test and *m*_o_ is the initial mass. All values reported are the average values of triplicates.

### Recyclability Test

To study the possibility of recycling lignin from the freeze-dried organogels, the highest loading of lignin-containing samples was used, i.e., PHB50-SKL50 and PHB50-HKL50. Triplicates of these freeze-dried organogels (∼30 mg) were transferred to vials and mixed with acetone. The vials were left under constant shaking for 24 h under ambient conditions. The mass was recorded, and the percentage of recycled lignin was calculated by eq 4.

4 $$Recycled\;lignin\left(\%\right)=\frac{m_{dry\;supernatant}}{m_{o}}\times 100$$

where *m*_dry supernatant_ is the dry mass of the acetone-extracted lignin and *m*_o_ is the initial mass of the freeze-dried organogel. All values reported are average values of triplicates. Based on eq 4, the fraction of lignin contained in the organogels was calculated.

## Results and Discussion

### Effect on Crystallinity

Freeze-dried PHB organogels in Cyrene containing varying amounts of softwood (SKL) and hardwood (HKL) kraft lignin were prepared to investigate the effect of the lignin type and content on the structure and properties of the resulting hybrid materials. The resulting chemical compositions were studied by FTIR spectroscopy (Figures 1 and S1). The addition of lignin into PHB enhances the –OH band at 3400 cm^–1^ and introduces new aromatic signals, characteristic of lignin.^42^ Specifically, the band at 1600 cm^–1^ corresponds to aromatic ring stretching, while the bands at 1511 cm^–1^ and 1421 cm^–1^ relate to aromatic skeletal vibrations.^42^ Additionally, the carbonyl C=O stretch at 1719 cm^–1^ in PHB decreases with the addition of either SKL and HKL, indicating hydrogen interactions between the phenolic –OH groups in lignin and the carbonyl in PHB.^43,44^ Interestingly, the double peaks at 680 cm^–1^ and 696 cm^–1^ diminish as more SKL or HKL is added to the organogel. These double peaks are attributed to out-of-plane C–H bending in CH_2_ and CH_3_ groups from the aliphatic backbone of PHB, which are indirectly linked to crystalline phase vibrations.^45^ These spectroscopic changes suggest that lignin incorporation, together with the organogel processing route, may influence the molecular packing and crystallization behavior of PHB.^44,46^

**Figure 1:**
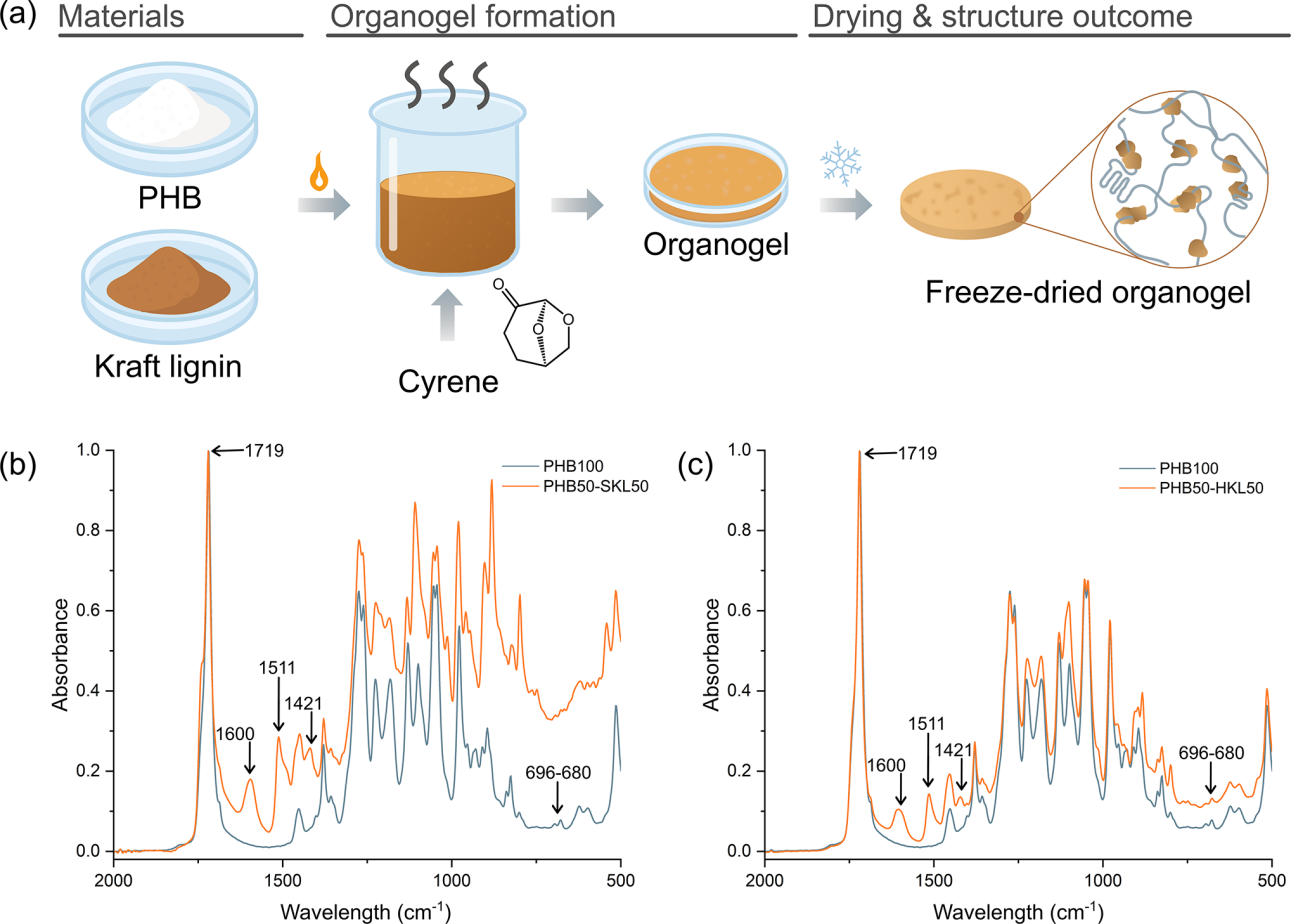
(a) Schematic representation of the process used to prepare lignin-containing PHB organogels, starting from the mixing of PHB with either softwood (SKL) or hardwood (HKL) kraft lignin and Cyrene to the drying of the organogel and final freeze-drying. Zoomed-in FTIR spectra of the PHB50-SKL50 (b) and PHB50-HKL50 organogels (c) with respect to PHB100. The arrows highlight the bands discussed in the main text related to lignin and PHB.

To further examine how these possible molecular interactions translate into differences in the bulk material structure, the morphology of the freeze-dried organogels was investigated using SEM. It was found that the process route had a significant impact on the obtained structures, as evident from the comparison of the PHB original and that of PHB100 (Figure S2). It seems that Cyrene promoted the formation of spherical particles that arise from droplet solidification during solvent removal and subsequent phase separation between PHB and the surrounding solution. Similar spherical particles can be observed for all lignin-containing organogels except for PHB50-SKL50, where the spherical particles disappeared (Figure S4). It could be because above this lignin content, the PHB phase becomes discontinuous, preventing the formation and stabilization of PHB-rich droplets (Figures 2 and S3). According to the literature, when PHB is dissolved in a polar aprotic solvent, such as dimethylformamide (DMF) or Cyrene, and subsequently precipitated by nonsolvent exchange or solvent removal, PHB-rich microdroplets form and solidify into spherical particles due to interfacial tension.^47^ Similar solvent-induced or emulsion-based processes have been employed to prepare PHB and poly­(3-hydroxy valerate) microparticles for coating and biomedical applications.^48^ The spherical morphology is therefore a consequence of polymer–solvent separation and rapid solidification, which minimizes surface energy and results in an optimal geometry. To give a better understanding of the relative sizes of these spheres, their diameters were measured manually (Figure S5). Although their size can be modulated based on the lignin content, no clear trend was observed for either formulation. This is another indirect indication that the presence of lignin and the process used to form these organogels affect their microstructure.

**Figure 2:**
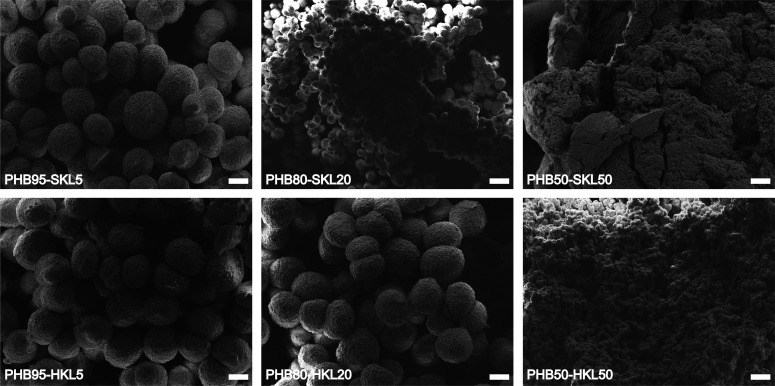
SEM images of the freeze-dried organogels containing 5, 20, and 50 wt % of SKL (top) and HKL (bottom). The scale bar for all images is 50 μm.

DSC measurements were performed to evaluate how the morphological changes caused by the organogel process and lignin incorporation affect the PHB crystallization and melting behavior (Table S1 and Figure S6). PHB100 exhibits a cold crystallization peak (*T*_cc_, i.e., the crystallization peak upon the second heating ramp) that is absent in the PHB original, indicating that rapid processing limits nucleation and promotes the formation of amorphous regions, a typical trait of slowly crystallizing polymers like PHB and PLA.^49,50^ This behavior suggests that Cyrene partially solvates and plasticizes PHB during gel formation, enabling chain reorganization and formation of a physically cross-linked network while simultaneously suppressing crystal perfection, consistent with the reduced crystallinity and cold-crystallization behavior discussed below. The PHB original displays sharp, well-separated double melting peaks (*T*_m1_ and *T*_m2_). This stems from the different interlamellar dimensions in the crystallites and differences in the polymer’s microstructure.^2,51^ In contrast, these peaks become broader and merge in lignin-containing samples, with the depression of peaks becoming more noticeable as the lignin content increases. This behavior, which is consistent with the *T*_cc_–*T*_m2_ relationship (Figure 3a), indicates increased crystal imperfections and higher thermal energy needed for reorganization. A systematic rise in *T*_g_ is also observed with increasing lignin content, with HKL having values slightly higher than those of SKL, suggesting a stronger restriction of chain mobility. This could be due to HKL’s higher polarity, enabling stronger hydrogen bonding and secondary interactions with PHB, as confirmed by FTIR spectroscopy (Figure 1).^27,29,52^ The disappearance of the crystallization peak (*T*_c_) at high lignin content indicates that crystallization during cooling is progressively hindered, leading to cold recrystallization during the second heating. Notably, the *T*_c_ peak and the corresponding crystallization enthalpy (Δ*H*_c_) vanish at lower lignin loadings for HKL-containing samples compared to SKL-containing ones, indicating a more pronounced suppression of crystallization during cooling in the presence of HKL. In contrast, the melting enthalpy (Δ*H*_m_) decreases consistently for both SKL- and HKL-containing samples, reflecting a reduction in the overall PHB mass fraction. Meanwhile, the cold-crystallization enthalpy (Δ*H*_cc_) increases upon lignin addition but remains constant within experimental error across lignin-containing samples, suggesting that the fraction of material reorganizing upon reheating is largely independent of lignin content. A pronounced decrease in the degree of crystallinity (*X*_c_^DSC^) is observed for both SKL- and HKL-containing organogels with an increasing lignin content (Figure 3b). At comparable lignin content, SKL-containing samples generally exhibit slightly higher crystallinity than their HKL counterparts, except for the highest lignin content. This trend suggests that HKL more effectively suppresses the final crystalline fraction of PHB at low-to-intermediate loadings. The slower gelation and incomplete network formation observed in HKL-containing organogels during processing further support the conclusion that HKL acts as a more effective crystallization inhibitor per unit mass of PHB compared to SKL.

**Figure 3:**
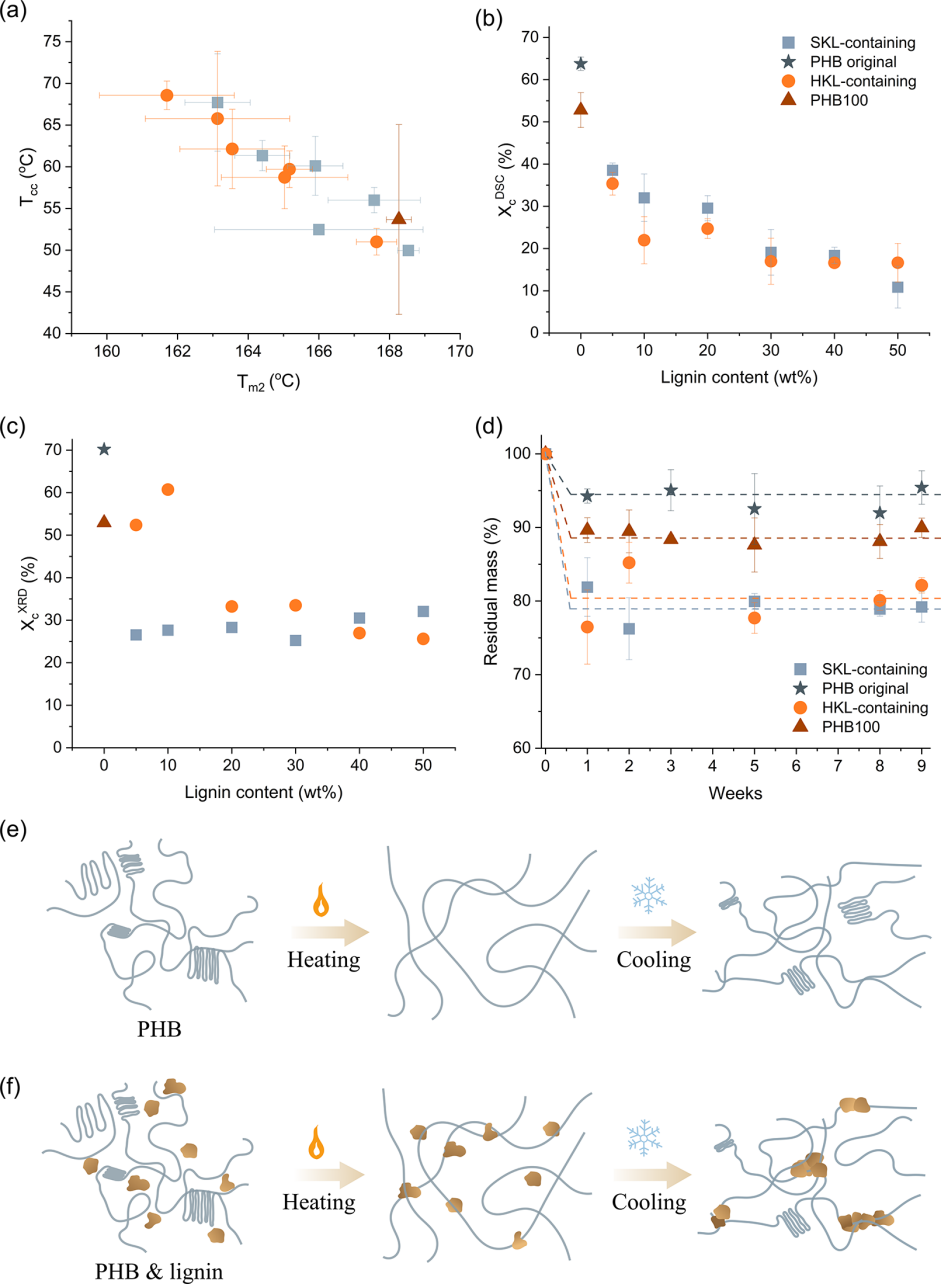
Physicochemical properties and water stability of the PHB organogel and its components. (a) *T*_cc_ against *T*_m2_. (b) DSC-derived *X*_c_ against the lignin content. (c) XRD-derived *X*_c_ against lignin content. (d) Residual mass after the water stability experiment. SKL- (gray squares), HKL-containing organogels (orange circles), PHB original (dark-gray star), and PHB100 (brown upward triangle). (e) Schematic representation of the disruption of the PHB crystalline domains during the cooling of PHB organogels by the presence of Cyrene alone. (f) Schematic representation of the disruption of the PHB crystalline domains during the cooling of PHB organogels by the presence of lignin. The dashed lines in (d) are used to guide the eye.

To further investigate the crystallinity of the freeze-dried organogels, we employed XRD. The crystallite size of PHB (*d*_020_) was estimated from the (020) reflection using the Debye–Scherrer equation (eq 2), as this peak at 13.5° represents the most intense diffraction feature of PHB (Table S2). A pronounced reduction in *d*_020_ is observed upon organogel formation, decreasing from approximately 25 nm for the PHB original to about 18 nm for PHB100.^41,53^ This indicates a clear disruption of crystal growth induced by the processing route. In contrast, lignin-containing samples do not exhibit a monotonic dependence of crystallite size on the lignin content. A noticeable difference between the SKL- and HKL-containing samples is apparent at the lowest lignin content, where the latter display a smaller crystallite size, while at higher lignin contents, the values converge within a narrow range. This, together with the earlier disappearance of *T*_c_ in DSC, suggests that HKL interferes more effectively with PHB crystal growth at the early stages of crystallization. At higher lignin contents, crystallization is strongly constrained for both systems, leading to comparable crystallite sizes, regardless of lignin type.

The lattice spacings and unit cell parameters were derived exclusively from the (020) and (110) reflections, as these peaks remained well-defined upon addition of lignin, whereas higher-order reflections became progressively broadened and indistinct due to the presence of lignin (Table S2 and Figure 4). The *d*-spacings and the corresponding lattice parameters (*a* and *b*) remain very close to those of the PHB original across all lignin-containing samples, with no systematic variations as a function of lignin content or type.^41,46^ This suggests that the orthorhombic crystal structure of PHB^40^ is largely preserved upon lignin incorporation and that lignin primarily affects the extent of crystallization rather than the crystal lattice itself. Consistent with this interpretation, the intensities of the characteristic PHB diffraction peaks decrease progressively with increasing lignin content, reflecting a reduction in overall crystallinity (Figure 4).^54,55^ The crystallinity trends obtained from DSC and XRD reveal similar qualitative behaviors but differ quantitatively at higher loadings (Figure 3b,c). At low lignin content, i.e., below 20 wt %, *X*_c_^XRD^ values are slightly higher than those obtained from DSC. However, above 20 wt % of lignin, the *X*_c_^XRD^ becomes relatively constant and remains systematically higher than the corresponding DSC values, whereas *X*_c_^DSC^ continues to decrease with increasing lignin content.^56^ These differences arise from how each technique probes the crystalline content. DSC measures the thermodynamically active crystalline fraction through melting and recrystallization enthalpies, making it sensitive to imperfect, constrained, or kinetically inaccessible crystals. In contrast, XRD probes the long-range crystalline order and can still detect small or imperfect crystallites that contribute weakly to the melting enthalpy. At high lignin loadings, the increasing amorphous fraction and lignin-induced confinement likely suppress crystal growth and perfection, reducing the DSC-detected crystalline fraction, while the remaining PHB crystallites retain sufficient long-range order to be detected by XRD. Similar discrepancies between DSC- and XRD-derived crystallinity have been reported for PHB/lignin systems and other polymer blends containing high fractions of amorphous fillers.^24^

**Figure 4:**
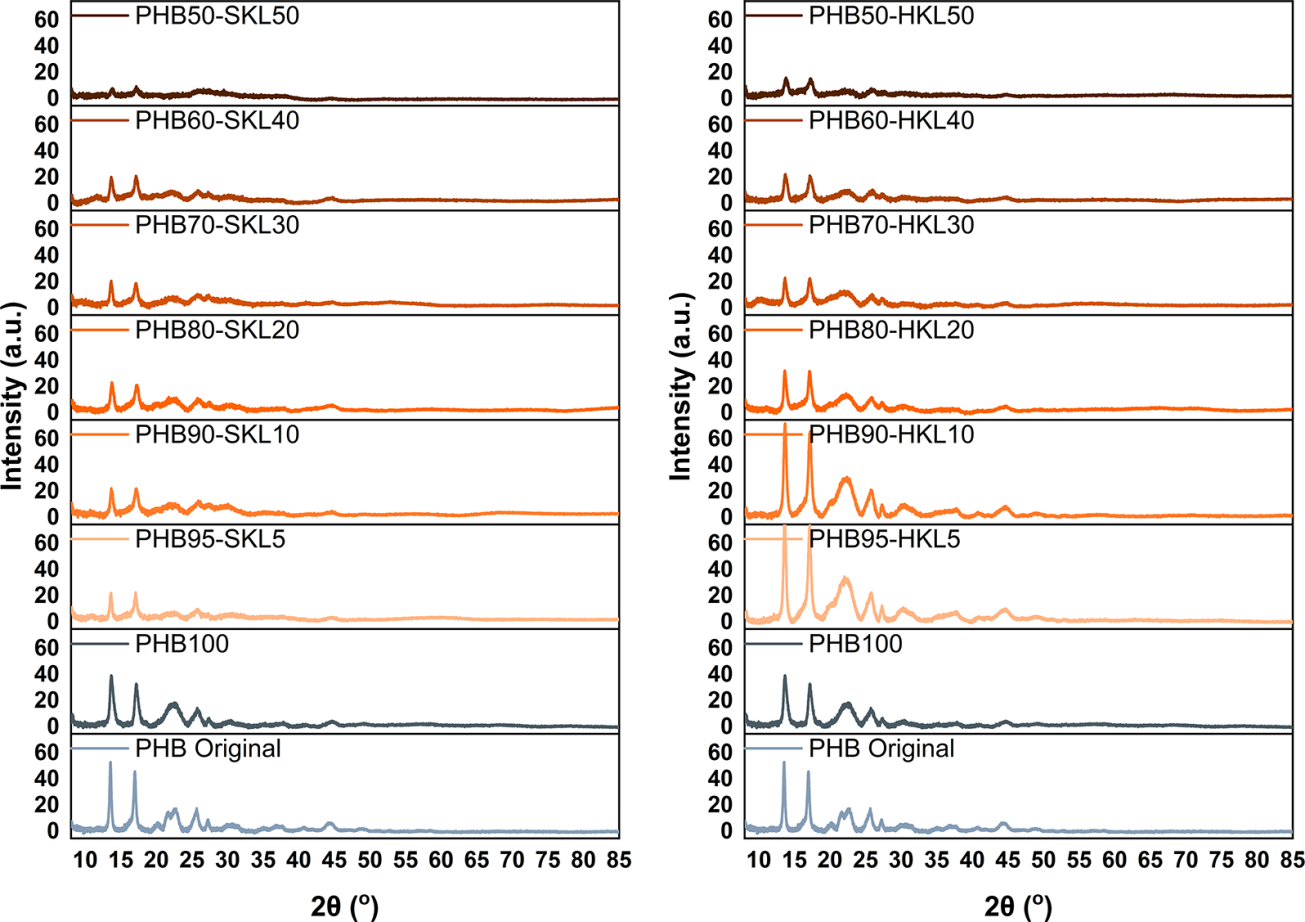
XRD spectra for the SKL- (left) and HKL-containing samples (right).

### Water-Stability Test

Water stability experiments were conducted to evaluate the impact of processing and lignin incorporation on mass loss over time (Figure 3d). This test was chosen as a degradation probe due to its experimental simplicity, short time scale, and sensitivity to changes in polymer crystallinity, making it suitable for comparison rather than comprehensive biodegradation testing. From the literature, it is expected that both acid- and base-catalyzed hydrolysis will result in improved degradation of PHB (within a few days) as the degradation occurs on crystalline domains, whereas at neutral pH, degradation occurs solely on the amorphous regions.^57^ Other studies related to enzyme-catalyzed hydrolysis^58,59^ and soil biodegradation studies^60^ have shown faster degradation of PHB. However, in this work, we investigated the degradation of PHB blends under neutral conditions, which were deemed more challenging.

A slight but consistent reduction in residual mass was observed for PHB100 compared to the PHB original, indicating that the Cyrene-mediated organogel formation increases hydrolytic susceptibility, consistent with the reduced crystallinity and altered crystal structure seen in DSC and XRD analyses. Adding lignin further increases the mass loss compared to systems without lignin. However, there was no statistically significant difference between SKL- and HKL-containing organogels, as both showed similar residual masses within experimental error throughout the hydrolysis. This suggests that although lignin addition improves water accessibility through crystallinity reduction and morphological disruption, the specific lignin type does not significantly change hydrolysis rates under the mild conditions used. The hydrolysis results support the idea that organogel processing and lignin addition influence the hydrolytic susceptibility, which is linked to the crystallinity of PHB mainly through structural changes rather than lignin-specific degradation pathways.

## Recyclability

To evaluate lignin’s role in inhibiting PHB crystallization and to demonstrate the circularity of the organogel-based process, the recyclability of lignin-containing organogels with the highest lignin content, i.e., 50 wt %, was examined. After extraction in acetone for 24 h, 42 ± 1% and 56 ± 2% of SKL and HKL, respectively, were obtained as a soluble fraction. Although hardwood lignin is more effective at suppressing crystallization, its higher extractability can be explained by its finer dispersion within the PHB matrix, which enhances solvent access despite stronger intermolecular interactions. In contrast, the more condensed and aggregation-prone structure of softwood lignin likely causes the formation of lignin-rich regions that are physically trapped within the PHB network, making them less extractable. In addition to dispersion effects, the higher extractability of HKL may also be influenced by differences in the lignin chemistry. HKL generally exhibits a lower degree of condensation and a higher abundance of polar functional groups compared with SKL, which may enhance compatibility with polar solvents such as acetone. SEM analysis showed that lignin extraction significantly changes the freeze-dried morphology (Figure S7). However, the resulting structures do not revert to those of samples originally prepared with similar lignin contents. This suggests that the morphology of the organogel remains kinetically trapped after lignin removal. While the HKL-containing samples retained partially collapsed spherical features, the SKL-containing samples showed no spherical shape, consistent with their original microstructure before extraction.

DSC analysis after extraction showed a sharp increase in *X*_c_, reaching about 60% for both SKL- and HKL-derived samples, matching that of the PHB original (Table 1). This increase is because of the removal of lignin-induced restrictions on PHB chain mobility, along with solvent-induced recrystallization during acetone extraction, which promotes lamellar thickening and crystal perfection.^61,62^ Notably, following lignin extraction, a clear crystallization exotherm during cooling, i.e., *T*_c_, was recovered, indicating that PHB chains regain sufficient mobility to crystallize during cooling, in contrast to the suppressed crystallization observed in the lignin-containing organogels before extraction. Overall, these findings demonstrate that lignin has a vital and reversible role in regulating PHB crystallization and that the organogel-based method allows partial recovery of lignin while producing a highly recrystallized PHB phase.

**Table 1:** Thermal Properties of the PHB Organogels before and after the Extraction of Lignin in Acetone

sample name	*T*_g_ (°C)^a^	T_m1_ (°C)^b^	T_m2_ (°C)^c^	ΔΗ_m_ (J/g)^d^	*T*_c_ (°C)^e^	ΔΗ_c_ (J/g)^f^	*T*_cc_ (°C)^g^	ΔΗ_cc_ (J/g)^h^	*X*_c_^DSC^ (%)[Table-fn t1fn9]
**PHB60-SKL40 before**	6 ± 0.3	151 ± 3	165 ± 1	50 ± 4	----	----	61 ± 2	31 ± 2	18 ± 2
**PHB60-SKL40 after**	4 ± 1	160 ± 2	170 ± 0.3	82 ± 5	64 ± 3	33 ± 2	49 ± 1	14 ± 1	60 ± 6
**PHB60-HKL40 before**	9 ± 1	147 ± 2	163 ± 2	55 ± 5	----	----	66 ± 8	37 ± 4	17 ± 1
**PHB60-HKL40 before**	3 ± 0.1	163 ± 1	170 ± 0.5	87 ± 2	69 ± 4	43 ± 2	46 ± 1	13 ± 0.1	58 ± 4

a Glass transition temperature.

b First melting peak of the second heating cycle.

c Second melting peak of the second heating cycle.

d Total enthalpy of melting.

e Crystallization temperature obtained from the cooling cycle.

f Enthalpy of crystallization.

g Cold crystallization temperature obtained from the second heating cycle.

h Enthalpy of cold crystallization.

j Crystallinity degree.

## Conclusions

PHB organogels prepared in the biomass-derived solvent Cyrene provide an effective route to tailor PHB crystallinity through solvent-mediated processing and lignin incorporation. DSC and XRD showed that organogel formation alone reduces crystallinity and induces cold crystallization, while the addition of lignin further suppressed crystallization during cooling and decreased the final crystalline fraction. Despite differences in gelation behavior, SKL- and HKL-containing organogels exhibited comparable crystallinity trends and hydrolytic stability under the conditions studied. However, small systematic differences were observed, including earlier *T*_c_ suppression and reduced crystallite size at low HKL loadings. Water stability experiments supported these structural findings by showing an increased mass loss for organogel-processed PHB relative to the original polymer. Finally, partial lignin recovery and the associated recrystallization of PHB upon solvent extraction demonstrated the reversibility and circular potential of the system, highlighting the link among processing-induced morphology, crystallinity, and material stability.

Future work should focus on quantifying lignin dispersion and interfacial interactions more directly (e.g., SAXS, rheology, solid-state NMR) and evaluating biodegradation under environmentally relevant conditions, such as by composting or in enzymatic media. In addition, the ability to incorporate high lignin contents while tuning crystallinity suggests potential application of these organogels as biobased porous materials, coatings, or scaffold-like structures where controlled hydrolytic stability is desirable. Finally, optimizing solvent recovery and lignin recycling efficiency could enable Cyrene-based PHB-lignin organogels as a scalable, circular route toward value-added biopolymer composites.

## Supplementary Material



## Notes

Additional DSC thermograms, XRD diffractograms, and crystal lattice parameters; FTIR spectra of PHB and lignin-containing organogels; SEM micrographs of the freeze-dried organogels; and images before and after lignin extraction (PDF)
