# Supplementary material for: Controlling the Properties of Poly(3-hydroxybutyrate) through Lignin-Containing Organogels
Source: Biomacromolecules. 2026 May 14;27(6):3869–78. doi: 10.1021/acs.biomac.6c00394 (PMC13250903; doi:10.1021/acs.biomac.6c00394)
Supplement: Supplementary file 1 [file bm6c00394_si_001.pdf]

Supplementary information

Controlling the properties of poly(3-hydroxybutyrate) through lignin-containing organogels

Alexandros E. Alexakis[1§], Unnimaya Thalakkale Veettil[1], Minna Hakkarainen[2], Mika. H. Sipponen[1]

Affiliations

[§] Corresponding author (email: alexandros.alexakis@su.se)

[1] Stockholm University, Department of Chemistry, 10691 Stockholm, Sweden

[2] KTH Royal Institute of Technology, Department of Fibre and Polymer Technology, Teknikringen 58, 100 44 Stockholm, Sweden

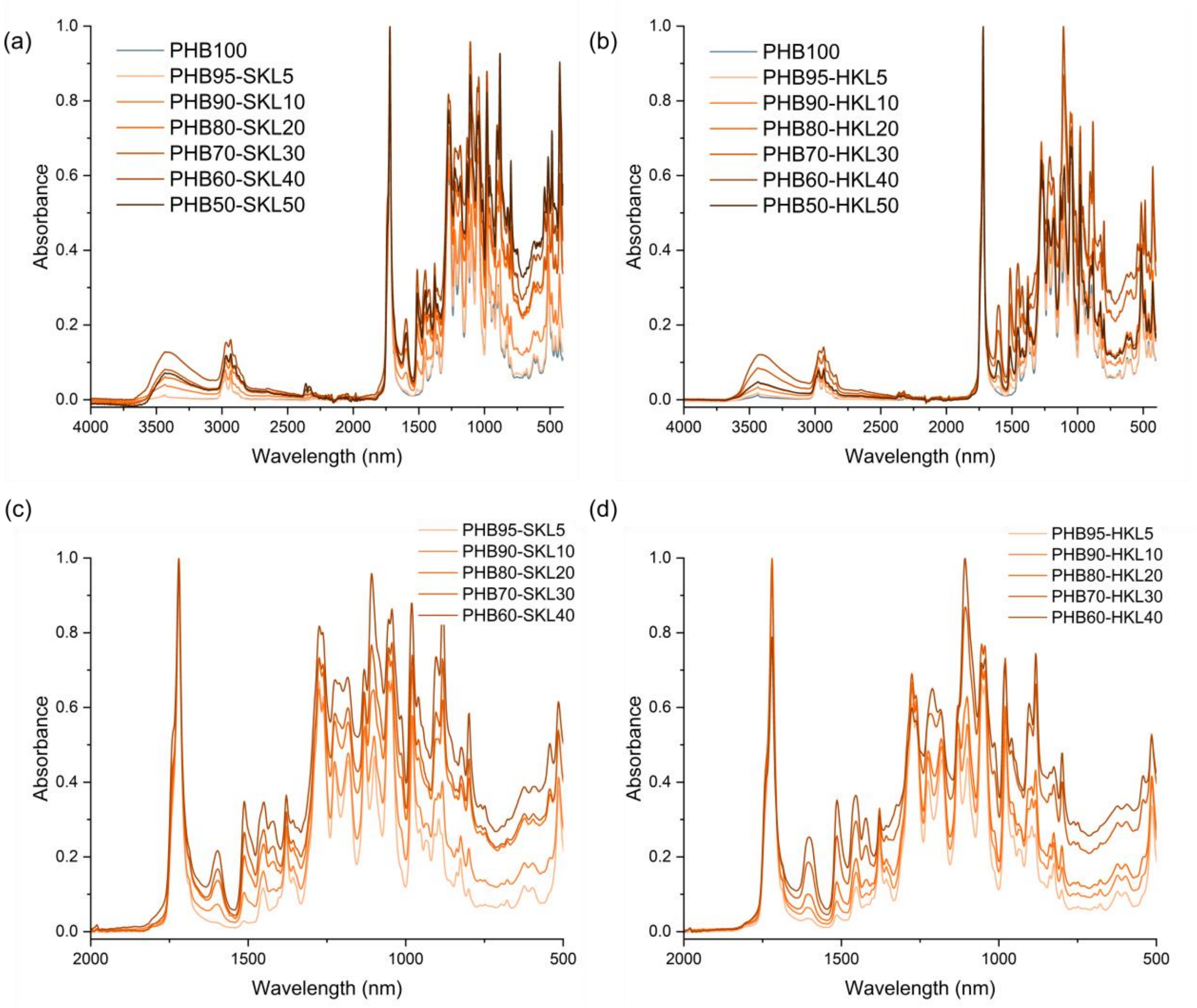

Figure S1. Full-range FTIR spectra of SKL- (a) and HKL-containing samples (b) in comparison with the PHB100 and their respective zoomed-in spectra (c) and (d).

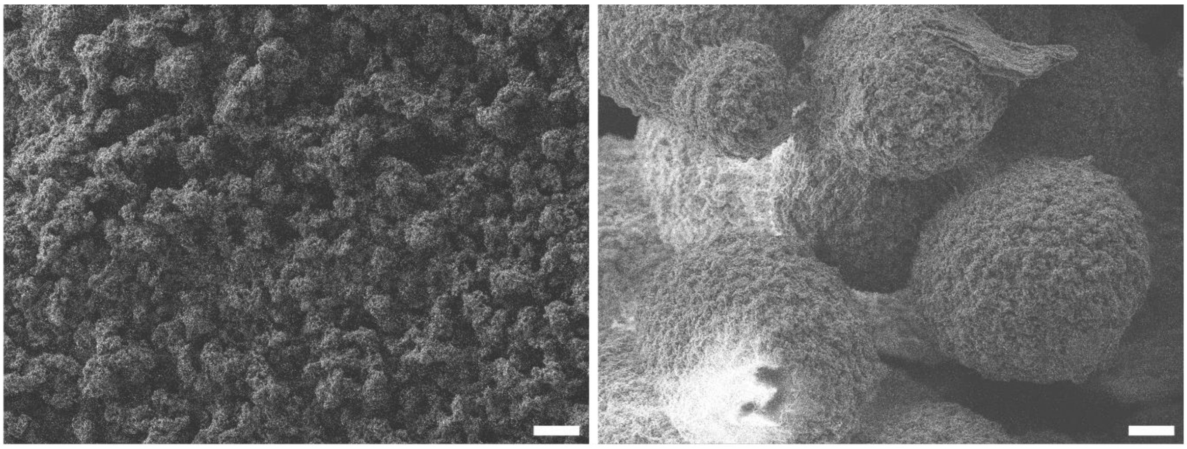

Figure S2. SEM images of the freeze-dried original PHB powder (left) and PHB100 (right). The scale bar for all images is 1 µm.

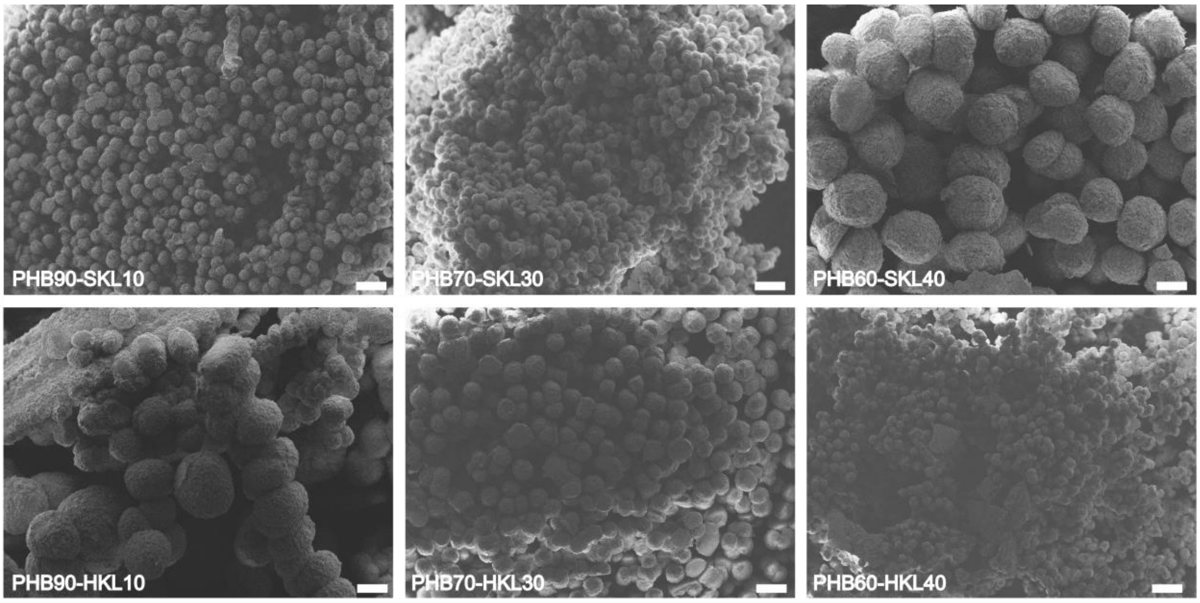

Figure S3. SEM images of the freeze-dried organogels containing 10 wt%, 30 wt%, and 40 wt% of SKL (top) and HKL (bottom). The scale bar for all images is 50 µm.

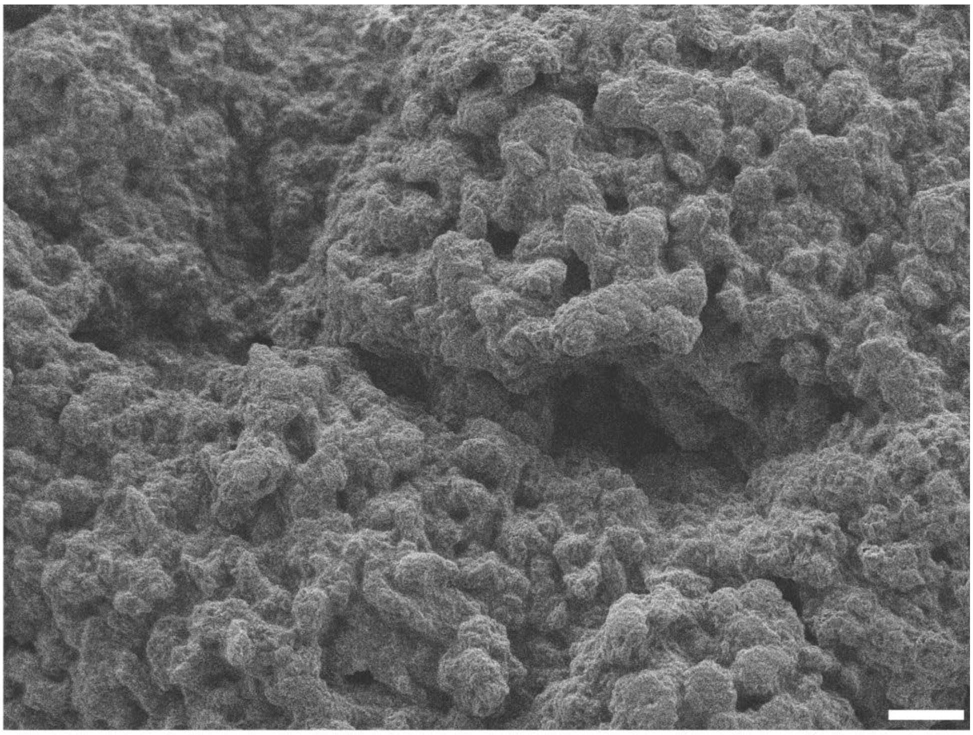

Figure S4. SEM image of PHB50-SKL50. The scale bar is 10 µm.

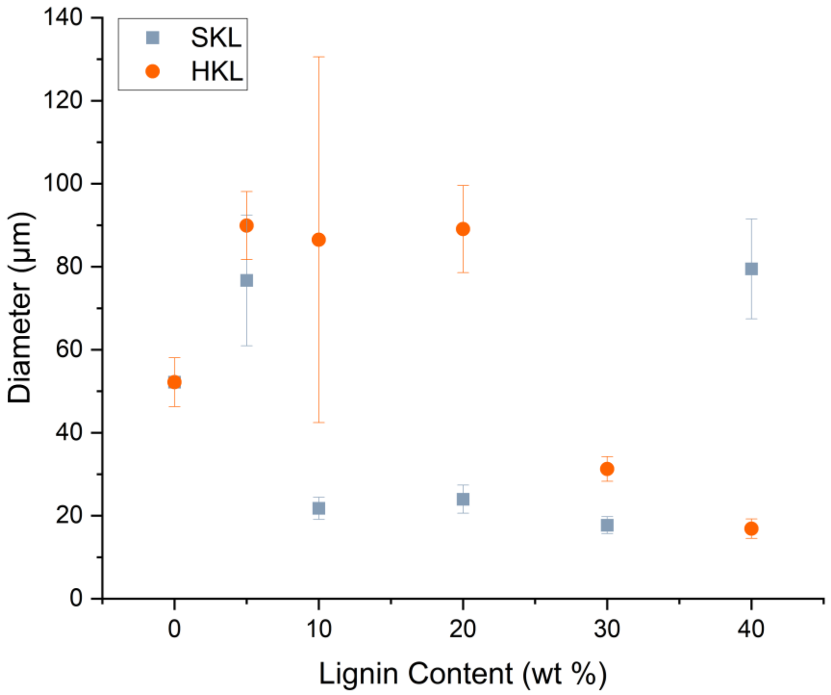

Figure S5. Diameter of the spherical structure observed in SEM for SKL- (blue squares) and HKL-containing samples (orange circles). The measurement was performed manually and averaged over 50 spheres.

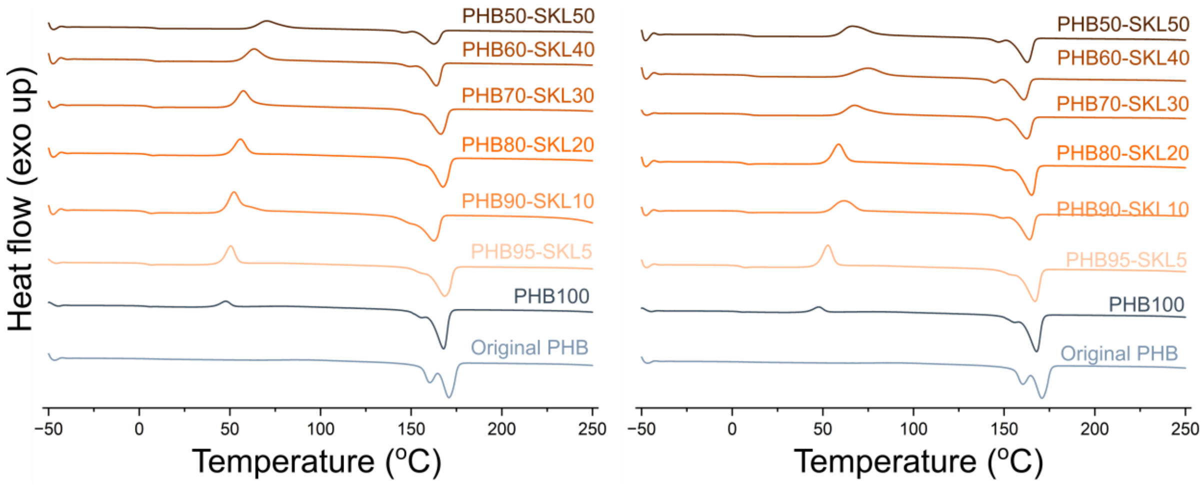

Figure S6. DSC thermograms of the freeze-dried organogels.

Table S1. Thermal properties of the PHB organogels.

**Sample Name**	**T_g (°C)**[a]	**T_{m1} (°C)**[b]	**T_{m2} (°C)**[c]	**ΔH_m (J/g)**[d]	**T_c (°C)**[e]	**ΔH_c (J/g)**[f]	**T_{cc} (°C)**[g]	**ΔH_{cc} (J/g)**[h]	**X_c^{DSC} (%)**[j]
**PHB original**	4±1	160±1	171±1	93±2	78±1	70±4	----	----	64±2
**PHB100**	3±0.4	156±0.1	168±0.4	83±4	62±1	46±4	54±11	6±2	53±4
**PHB95-SKL5**	3±0.1	157±3	169±0.3	81±3	64±2	17±2	50±1	27±2	39±2
**PHB90-SKL10**	4±1	156±5	166±3	78±4	66±5	10±5	53±1	35±9	32±6
**PHB80-SKL20**	4±2	156±3	167±1	68±3	64±6	3±1	56±2	32±1	30±3
**PHB70-SKL30**	6±1	152±3	166±1	56±6	65±0.2	2±1	60±4	35±4	19±5
**PHB60-SKL40**	6±0.3	151±3	165±1	50±4	----	----	61±2	31±2	18±2
**PHB50-SKL50**	9±1	148±4	163±1	33±7	----	----	68±6	22±4	10±5
**PHB95-HKL5**	3±0.3	154±1	168±1	80±2	61±5	11±5	51±2	32±4	36±3
**PHB90-HKL10**	5±1	149±1	165±2	62±8	69±6	3±2	59±4	33±2	22±6
**PHB80-HKL20**	6±1	152±3	165±1	62±4	73±15	2±1	60±2	32±2	25±2
**PHB70-HKL30**	8±2	149±3	164±2	57±7	----	----	62±5	38±2	17±6
**PHB60-HKL40**	9±1	147±2	163±2	55±5	----	----	66±8	37±4	17±1
**PHB50-HKL50**	9±0.6	145±3	162±2	50±6	----	----	69±2	34±3	17±5

[a] Glass transition temperature. [b] First melting peak of the second heating cycle. [c] Second melting peak of the second heating cycle. [d] Total enthalpy of melting. [e] Crystallization temperature obtained from the cooling cycle. [f] Enthalpy of crystallization. [g] Cold crystallization temperature obtained from the second heating cycle. [h] Enthalpy of cold crystallization. [j] Crystallinity degree calculated by equation 1. The ± denotes the standard deviation between three replicates.

Table S2. Lattice spacing and crystal lattice parameters calculated from the XRD results.

Sample Name	d_{020} (nm)	Lattice spacing (Å)		Crystal lattice parameters (Å)	
		$d_{(020)}$	$d_{(110)}$	a	b
**PHB original**	24.81	6.54	5.23	5.70	13.08
**PHB100**	17.46	6.51	5.19	5.66	13.02
**PHB95-SKL5**	21.78	6.51	5.19	5.66	13.02
**PHB90-SKL10**	16.18	6.48	5.17	5.64	12.96
**PHB80-SKL20**	14.54	6.44	5.14	5.61	12.88
**PHB70-SKL30**	18.82	6.50	5.18	5.65	13.00
**PHB60-SKL40**	16.15	6.49	5.17	5.64	12.98
**PHB50-SKL50**	14.63	6.43	5.14	5.61	12.86
**PHB95-HKL5**	15.44	6.44	5.15	5.62	12.88
**PHB90-HKL10**	12.73	6.49	5.19	5.66	12.98
**PHB80-HKL20**	16.36	6.49	5.18	5.65	12.98
**PHB70-HKL30**	15.98	6.48	5.17	5.64	12.96
**PHB60-HKL40**	13.65	6.45	5.15	5.62	12.90
**PHB50-HKL50**	12.93	6.40	5.13	5.60	12.80

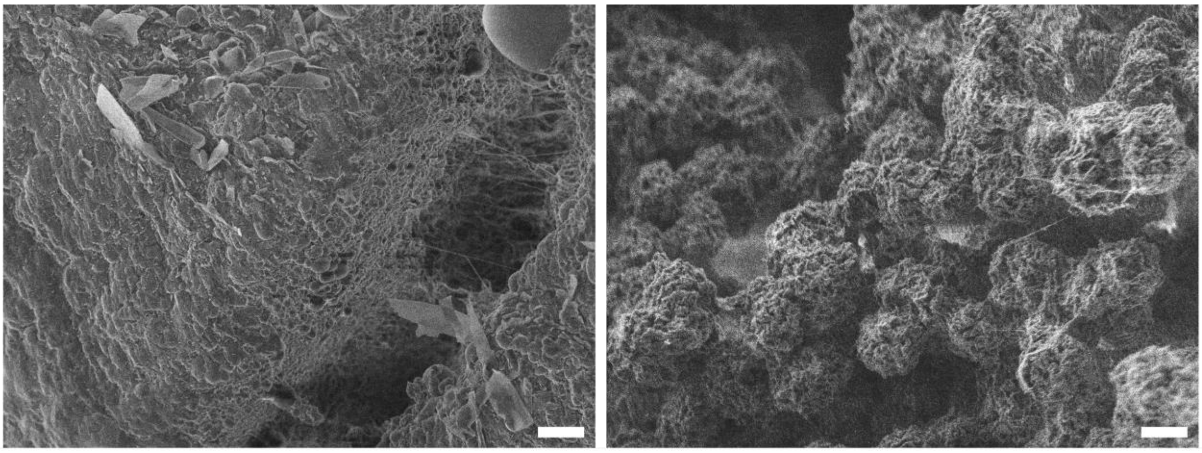

Figure S7. SEM of the SKL- (left) and HKL-containing (right) organogels after the extraction of lignin in acetone.
